# Biological predictors of survival in stage II colorectal cancer

**DOI:** 10.3892/mco.2013.126

**Published:** 2013-05-20

**Authors:** YOSHITAKE UEDA, KAZUHIRO YASUDA, MASAFUMI INOMATA, NORIO SHIRAISHI, SHIGEO YOKOYAMA, SEIGO KITANO

**Affiliations:** 1 Departments of Gastroenterological Surgery, Oita University, Yufu, Oita 879-5593, Japan;; 2 Pathology, Faculty of Medicine, Oita University, Yufu, Oita 879-5593, Japan;; 3 Oita University, Yufu, Oita 879-5593, Japan

**Keywords:** colorectal cancer, prognostic factor, lymphatic invasion, lymph node micrometastasis

## Abstract

The routine use of postoperative adjuvant chemotherapy in patients with stage II colorectal cancer is not recommended. However, the incidence of tumor recurrence or distant metastasis in these patients is reported to be 25–35%. The identification of high-risk patients with stage II colorectal cancer remains difficult. Therefore, the aim of this study was to determine the risk factors that may help identify stage II colorectal cancer patients with unfavorable prognosis. Paraffin-embedded tissue samples from 109 patients with stage II colorectal cancer following curative operation were analyzed. Thirteen clinicopathological variables and 5 biological markers were assessed using immunohistochemistry, including p53 (tumor suppressor gene), CD10 (tumor invasion marker), CD34 (angiogenic marker), Ki-67 (cell proliferation index) and CAM 5.2 (marker of lymph node micrometastasis) and investigated for associations with disease-specific survival. Univariate analysis revealed bowel obstruction, lymph node micrometastasis and lymphatic invasion (P<0.01) to be highly significant factors for determining the 5-year disease-specific survival. By contrast, the multivariate analysis revealed lymph node micrometastasis and lymphatic invasion to be independent prognostic factors. Stage II colorectal cancer patients with lymph node micrometastasis and lymphatic invasion may therefore be suitable candidates for adjuvant chemotherapy to improve prognosis.

## Introduction

Lymph node metastasis is the most powerful predictor of recurrence or survival in patients with colorectal cancer. Although the majority of patients with node-negative colorectal cancer are potentially cured with surgery alone, ≤25% are likely to present with recurrence and succumb to the disease (1). Identifying high-risk patients with stage II colorectal cancer is important for determining which patients may benefit the most from adjuvant chemotherapy.

The effect of clinicopathological factors on recurrence and survival following curative resection has been the subject of several studies and numerous clinicopathological factors have been suggested as prognostic indicators for colorectal cancer. It is important to determine which of these factors affect the risk of recurrence in the node-negative colorectal cancer patients and which factors should be prospectively applied in the routine clinicopathological evaluation of colorectal cancer.

With the recent developments in immunohistochemistry and molecular biology, several biological markers have been extensively investigated (2–12). In this study, the expression of several biological markers, including p53, CD10, CD34 and Ki-67, which are strongly suspected of playing a significant role in tumor progression, was evaluated. Additionally, we focused on lymph node micrometastasis, which is easily detected by antibody CAM 5.2.

The aim of this study was to conduct a multivariate analysis of the prognostic impact of a wide range of clinicopathological and biological variables in patients with stage II colorectal cancer.

## Materials and methods

### Patients

We reviewed all the patients who underwent curative resection for stage II colorectal cancer at the Department of Gastroenterological Surgery, Oita University Hospital, between 1984 and 2002. Patients who had received preoperative chemoradiation for locally advanced lower rectal cancer were excluded from this cohort study. Ultimately, 109 patients (61 males and 48 females; average age, 67 years; range, 31–89 years) were enrolled and the tumors were diagnosed as clinical stage T3, N0 and M0.

### Evaluation

The survival analysis was performed for the following clinicopathological factors: age, gender, location of tumor (right vs. left colon vs. rectum), number of resected lymph nodes (0–11 vs. ≥12), bowel obstruction (absent vs. present), tumor size (0–4 vs. >4 cm), depth of tumor invasion (subserosa vs. serosa), tumor differentiation (high vs. moderate vs. mucinous), lymphatic invasion (absent or mild vs. moderate or severe), venous invasion (absent vs. present), tumor budding (absent vs. present) (13), peritumoral lymphocytes (inconspicuous vs. conspicuous) (14) and tumor growth pattern (expansive vs. infiltrative) (14). Furthermore, 5 biological markers were asessed using immunohistochemistry, including p53 (tumor suppressor gene), CD10 (tumor invasion marker), CD34 (angiogenic marker), Ki-67 (cell proliferation index) and CAM 5.2 (marker of lymph node micrometastasis). Tumor budding is defined as an isolated single cancer cell or a cluster composed of <5 cancer cells observed in the stroma of the actively invasive region. A count of 0–9 per field was considered as absent and a count of ≥10 was regarded as present, based on the results of a previous study (13). The characteristics of peritumoral lymphocytes (inconspicuous vs. conspicuous) and the tumor growth pattern (expansive vs. infiltrative) were assessed strictly according to the criteria originally described by Jass *et al* (14).

### Immunohistochemistry

Resected tumors from each of the 109 patients were fixed in 10% formalin solution and embedded in paraffin. Representative tissue sections, each containing the deepest site of cancer invasion, were cut at 4-*μ*m. As regards the lymph node specimens, one 3-*μ*m section was obtained for hematoxylin and eosin staining and five serial 6-*μ*m sections for immunohistochemical staining. The avidin-biotin peroxidase complex method was used for detection of the five monoclonal antibodies in deparaffinized and rehydrated tissue sections. Antigen retrieval was performed by placing the sample in a microwave oven at 95°C for 40 min, followed by cooling for 30 min to room temperature, except for CD34 and CAM 5.2. CAM 5.2 sections were trypsinized with 0.1% calcium chloride solution. p53, Ki-67, CD10 and CD34 were incubated for 2 h at room temperature and CAM 5.2 was incubated overnight at 4°C. The slides were then incubated for 30 min with EnVision™ peroxidase mouse system (DAKO Corporation, Carpinteria, CA, USA). The color reaction product was developed with diaminobenzidine tetrahydrochloride (DAB; Sigma Chemical Co., St. Louis, MO, USA) as the chromogen for 5 min. Using a light microscope, a visual grading system was used based on the number of positively stained nuclei of the cancer cells in each tissue sample.

p53 slides were scored according to the percentage of positive tumor nuclei as follows: positive, ≥10% of the nuclei stained; negative, <10% of the nuclei stained (3). For Ki-67 immunoreactivity, staining was considered positive at >60% (9). Tumor positivity for CD10 was evaluated using a predetermined cut-off of 5% (positive, >5% tumor cell staining) according to a previous study (10). CD34 slides were classified according to the microvessel count. After scanning the highly vascularized areas, we selected three areas exhibiting the most prominent neovascularization. A microvessel count was performed on a ×400 field (×40 objective and ×10 ocular) and the average count from the three areas was calculated (15). Patients were divided into those with a microvessel count of 0–50 and those with a microvessel count of >50. As regards metastatic lymph nodes, patients were divided into two groups according to a previous study (16), those with micrometastasis in ≤3 lymph nodes and those with micro-metastasis in ≥4 lymph nodes. Written informed consent was obtained from all the patients and this study was approved by the local Ethics Committee.

### Statistical analysis

Data were statistically analyzed using SPSS statistical software (Statistical Package for Social Sciences). Univariate disease-specific survival analysis was performed using the Kaplan-Meier method and the difference was evaluated by the log-rank test. Multivariate analysis was performed using the Cox proportional hazards model. P<0.05 was considered to indicate a statistically significant difference.

## Results

### Factors affecting patient survival

The study included a total of 61 males and 48 females, with an average age of 67 years (range, 31–89 years). The median follow-up period for the survivors was 5.7 years (range, 1.7–11 years). At the time of analysis, 87 patients were free of disease, 7 were alive with disease, 15 had succumbed to the disease and 5 patients had succumbed due to other causes. Twenty-two patients developed recurrence or distant metastasis. Of these, 12 had liver metastases, 7 had local recurrence and 3 had lung metastasis. The 5-year disease-specific survival rate of patients with stage II colorectal cancer was 86.2%.

**Figure 1:**
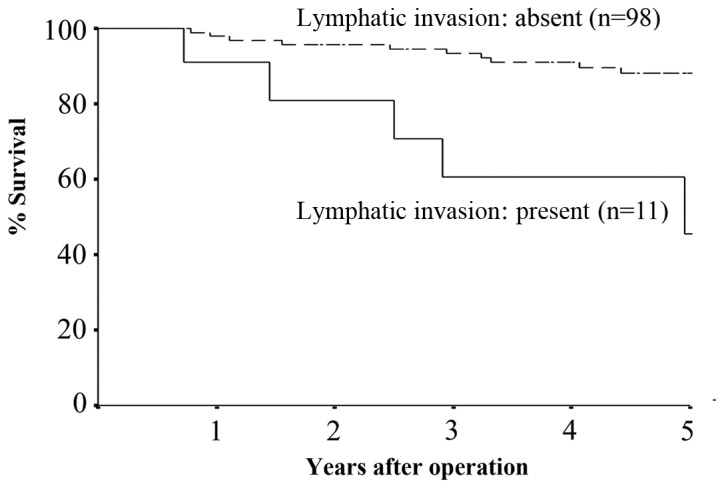
The 5-year disease-specific survival rate for the group of patients with lymphatic invasion was significantly lower compared to that for the group without lymphatic invasion (55 vs. 90%, P<0.01).

**Figure 2:**
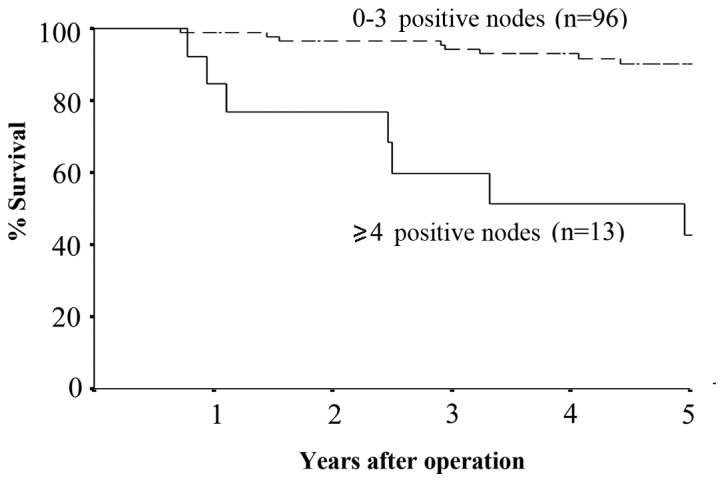
The 5-year disease-specific survival rate for the group of patients with ≥4 micrometastases was significantly lower compared to that for the group with 0–3 micrometastases (46 vs. 92%, P<0.01).

**Figure 3:**
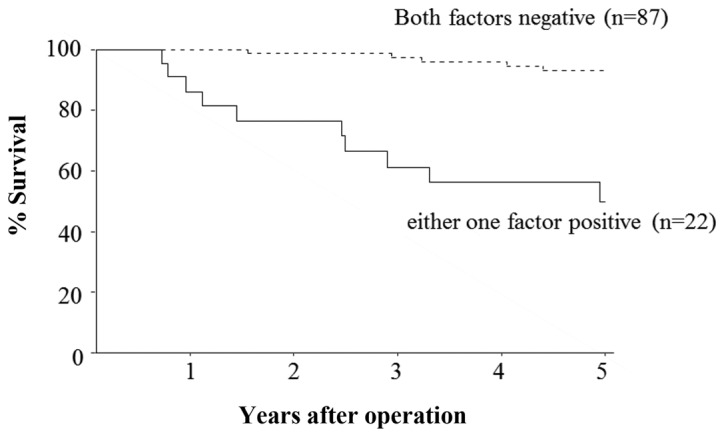
Lymphatic invasion and micrometastasis: the 5-year disease-specific survival rate for the group of patients with either one positive factor was significantly lower compared to that for the group with both factors negative (55 vs. 94%, P<0.01).

In the univariate analysis, bowel obstruction, lymph node micrometastasis and lymphatic invasion (P<0.01) were significant factors for determining the 5-year disease-specific survival (Table I). When all of these factors were included as independent variables in a Cox proportional hazards model, the presence of lymphatic invasion was the most powerful negative predictor of survival [hazard ratio (HR), 4.091; P=0.006], followed by lymph node micrometastasis (HR, 3.704; P= 0.011) (Table II). The 5-year disease-specific survival rate was significantly lower for the group of patients with moderate to severe presence of lymphatic invasion compared to that for the group with absent to mild presence of lymphatic invasion (55 vs. 90%, P<0.01) (Fig. 1). Similarly, the 5-year disease-specific survival rate was significantly lower for the group with ≥4 positive micrometastatic nodes compared to that for the group with 0–3 positive micrometastatic nodes (46 vs. 92%, P<0.01) (Fig. 2). When a combination of two factors, lymphatic invasion and micrometastasis was examined, the 5-year disease-specific survival rate for the group of patients with either one positive factor was significantly lower compared to that for the group with both factors negative (55 vs. 94%, P<0.01) (Fig. 3).

## Discussion

In the present study, 20% of the stage II colorectal cancer patients presented with tumor recurrence or distant metastasis during follow-up, after curative resection. A multivariate analysis allowed us to define a subgroup of patients at high risk of recurrence, which included those with lymph node micrometastasis and those with lymphatic invasion. In addition, these factors were significantly associated with the prognosis of stage II colorectal cancer patients.

Recent advances in immunohistochemistry and molecular biology suggest that molecular changes of the primary tumor may serve as prognostic indicators for individual patients. Several studies have attempted to identify the prognostic biomarkers in patients with stage II or node-negative colorectal cancer (2–7, 12,17,18).

Although several studies have been conducted on lymph node micrometastasis in patients with colorectal cancer, the significance of the presence of lymph node micrometastasis has been a subject of debate (16,18–21). Yasuda *et al* (16) reported that micrometastasis in ≥4 lymph nodes and micrometastasis to N2 or higher nodes were significantly correlated with postoperative recurrence and prognosis in stage II colorectal cancer patients. Bukholm *et al* (21) reported that the presence of isolated tumor cells in the mesenteric lymph nodes was independently associated with reduced relative survival in patients with stage II colon cancer. Our study also demonstrated that the number of lymph node micrometastases was a more powerful indicator than the presence and level of lymph node micrometastasis. Therefore, it is helpful to investigate the number of lymph node micrometastases with immunohistochemistry in stage II colorectal cancer patients.

The aim of adjuvant chemotherapy is the destruction of microscopic metastases that may already be present and the reduction of the risk of recurrence. Postoperative chemotherapy for stage III colorectal cancer patients has been shown to improve prognosis and is recommended as standard therapy (22,23). However, the value of adjuvant chemotherapy for patients with stage II colorectal cancer is controversial (24,25). The International Multicentre Pooled Analysis of B2 Cancer Trials (IMPACT B2) (26) and the meta-analysis reported by Figueredo *et al* (27) did not demonstrate any improvement in prognosis of stage II colon cancer patients treated with adjuvant chemotherapy. However, the QUASAR study demonstrated a significantly reduced recurrence rate and improved survival of patients with stage II colorectal cancer in favour of the adjuvant chemotherapy arm (28).

Although several large studies have investigated the subject of adjuvant chemotherapy for stage II colorectal cancer patients, the use of adjuvant chemotherapy for all stage II colorectal cancer patients may be inappropriate and expensive (29). Therefore, there is an increasing need for accurate stratification of stage II colorectal cancer patients in order to identify those at high-risk of recurrence who may benefit from adjuvant chemotherapy.

Our data suggest that two factors, lymph node micrometastasis and lymphatic invasion, should be included in the high-risk group of patients with stage II colorectal cancer. Sirop *et al* (30) reported improved outcomes of micrometastasis after being considered as high-risk disease and treated with chemotherapy in their pilot study. These results suggest a trend in favour of adjuvant chemotherapy in stage II colorectal cancer patients with high-risk factors.

In conclusion, we demonstrated that each of the two factors investigated, lymph node micrometastasis and lymphatic invasion, carries independent prognostic significance with respect to the 5-year disease-specific survival rates of patients with stage II colorectal cancer. This finding may be useful in identifying the high-risk patients for recurrence or metastasis among stage II colorectal cancer patients. We recommend that stage II colorectal cancer patients with lymph node micrometastasis and lymphatic invasion be evaluated for the benefit of adjuvant chemotherapy in the future, through further prospective randomized control studies.

## Figures and Tables

**Table I:** Univariate analysis for the 5-year disease-specific survival in patients with stage II colorectal cancer.

Factors	No. of patients	5-year survival rate (%)	P-value
Age (years)			
0–70	73	79	NS
≥71	36	81	
Gender			
Male	61	85	NS
Female	48	88	
Location of tumor			
Right colon	21	95	NS
Left colon	52	82	
Rectum	36	68	
No. of resected lymph nodes			
0–11	43	81	NS
≥12	66	79	
Bowel obstruction			
Absent	99	83	<0.01
Present	10	50	
Tumor size (cm)			
0–4	29	90	NS
>4	80	76	
Depth of tumor invasion			
T3	80	90	NS
T4	29	76	
Differentiation			
High	68	84	NS
Moderate	36	75	
Poor/mucinous	5	60	
Lymphatic invasion			
Absent, mild	98	90	<0.01
Moderate, severe	11	55	
Venous invasion			
Absent	80	89	NS
Present	29	79	
Tumor budding			
Absent	70	84	NS
Present	39	90	
Peritumoral lymphocytes			
Inconspicuous	40	83	NS
Conspicuous	69	88	
Tumor growth pattern			
Expansive	30	91	NS
Infiltrative	79	84	
p53			
Negative	49	84	NS
Positive	60	88	
CD10			
Negative	70	83	NS
Positive	39	92	
Angiogenesis (microvessel count)			
0–50	90	80	NS
>50	19	79	
Ki-67 index			
Sparse	94	78	NS
Diffuse	15	93	
Lymph node micrometastasis			
0–3 positive nodes	96	92	<0.01
≥4 positive nodes	13	46	

NS, non-significant.

**Table II:** Multivariate analysis for the 5-year disease-specific survival in patients with stage II colorectal cancer.

Factors	HR	95% CI	P-value
Lymphatic invasion	4.091	1.376–12.165	0.006
Lymph node micrometastasis	3.704	1.458–9.406	0.011

HR, hazard ratio; CI, confidence interval.
